# Publisher Correction to *Hepatology, Medicine and Policy*: Articles with DOIs 10.1186/s41124-017-0024-1, 10.1186/s41124-017-0025-0, 10.1186/s41124-017-0026-z and 10.1186/s41124-017-0027-y

**DOI:** 10.1186/s41124-018-0038-3

**Published:** 2018-09-14

**Authors:** BioMed Central

**Affiliations:** https://www.biomedcentral.com/

## Correction

The metadata in the HTML format of the below original articles [1–4] were published with an incorrect cover date. The correct cover date is December 2017. The metadata of the original articles have been corrected. This does not alter the text of the original articles.

The publisher apologizes for this processing error.
